# New Appliances and Things Medical

**Published:** 1908-08-01

**Authors:** 


### NEW APPLIANCES & THINGS MEDICAL.

### "4711" EAU DE COLOGNE FOR THE SUMMER.

The addition of a few drops of eau de Cologne to the water we use for toilet purposes in summer is regarded by most of us almost as a necessity when once it has been tried. Not only is the effect delightfully refreshing to the skin, but it also helps to allay the irritation caused by insect bites. In some cases when eau de Cologne is used very plentifully, it actually serves as a preventive. We are accustomed to regard eau de Cologne as indispensable in the sick room, but during a long journey we learn quickly to appreciate its use to the full. Nothing so quickly removes the traces of dust from the face, and with it the sense of fatigue and exhaustion. "4711" eau de Cologne possesses all the good qualities of perfumes of its class, but it is free from any unpleasant stale smell clinging after scent, which is very noticeable in some cases. It is increasingly popular, and can be obtained wherever good perfumes are sold.

